# Advance care planning with patients on hemodialysis: an implementation study

**DOI:** 10.1186/s12904-019-0437-2

**Published:** 2019-07-26

**Authors:** Sarah L. Goff, Mark L. Unruh, Jamie Klingensmith, Nwamaka D. Eneanya, Casey Garvey, Michael J. Germain, Lewis M. Cohen

**Affiliations:** 1 University of Massachusetts Medical School-Baystate, 759 Chestnut St., Springfield, MA 01199 USA; 2 Present Address: School of Public Health and Health Sciences, University of Massachusetts-Amherst, Amherst, MA 01002 USA; 3 School of Medicine, University of New Mexico, Albuquerque, NM 87131 USA; 4 Present Address: Perelman School of Medicine, University of Pennsylvania, Philadelphia, PA 19104 USA; 5 Massachusetts General Hospital, 55 Fruit St., Boston, MA 02114 USA; 6 Northeastern University, 360 Huntington Ave, Boston, MA 02115 USA

**Keywords:** Hemodialysis, End-of-life, Advance care planning, Shared decision-making, Intervention, Implementation

## Abstract

**Background:**

Patients with end-stage kidney disease (ESKD) on hemodialysis have limited life expectancy, yet their palliative care needs often go unmet. The aim of this study was to identify barriers and facilitators for implementation of “Shared Decision Making and Renal Supportive Care” (SDM-RSC), an intervention to improve advance care planning (ACP) for patients with ESKD on hemodialysis.

**Methods:**

The Consolidated Framework for Implementation Research (CFIR) was the organizing framework for this study. CFIR is a theory-based implementation framework consisting of five domains (Intervention Characteristics, Inner Setting, Outer Setting, Characteristics of Individuals, and Process), each of which has associated constructs. Potential barriers and facilitators to implementation of the SDM-RSC intervention were identified through observation of study procedures, surveys of social workers nephrologists, study participants, and family members, and assessment of intervention fidelity.

**Results:**

Twenty-nine nephrologists and 24 social workers, representing 18 outpatient dialysis units in Massachusetts (*n* = 10) and New Mexico (*n* = 8), were trained to conduct SDM-RSC intervention sessions. A total of 102 of 125 patient enrolled in the study received the intervention; 40 had family members present. Potential barriers and facilitators to implementation of the SDM-RSC intervention were identified in each of the five CFIR domains. Barriers included complexity of the intervention; challenges to meeting with patients on non-dialysis days; difficulties scheduling intervention sessions due to nephrologists’ and social workers’ caseloads; perceived need for local policy change regarding ACP; perceived need for additional ACP training for social workers and nephrologists; and lack of endorsement of the intervention by some staff members. Facilitators included: training for social workers, national dialysis chain leadership engagement and the institution of social worker/nephrologist clinic champions.

**Conclusions:**

ACP for patients on hemodialysis can have a positive impact on end-of-life outcomes for patients and their families but does not take place routinely. The barriers to effective implementation of interventions to improve ACP identified in this study might be addressed by: adapting the intervention for local contexts with input from clinicians, dialysis staff, patients and families; providing nephrologists and social workers additional training prior to delivering the intervention; and developing policy that routinizes ACP for hemodialysis patients.

**Trial registration:**

Clinicaltrials.gov NCT02405312. Registered 04/01/2015.

**Electronic supplementary material:**

The online version of this article (10.1186/s12904-019-0437-2) contains supplementary material, which is available to authorized users.

## Background

The estimated 458,000 people receiving hemodialysis for end stage kidney disease (ESKD) annually in the United States face one-year mortality rates of approximately 20 to 25% and five-year mortality rates of 35% [1]. When compared to other common life-limiting illnesses, more patients die from ESKD-related causes than from breast, prostate, or metastatic colon cancer every year [2, 3]. Physical and mental health comorbidities associated with the diseases that commonly cause ESKD and morbidity associated with hemodialysis itself affect quality of life (QoL): pain, visual impairment, and decreased mobility due to amputation related to diabetes and loss of autonomy, fatigue, dietary restrictions, sleep disorders, and depression associated with hemodialysis [4].The multiple threats to QoL make palliative care an important consideration for patients with ESKD on hemodialysis, but their palliative care needs often go unmet [5, 6].

National and international efforts to improve palliative care for patients on hemodialysis have included interventions to increase the use of advance care planning (ACP) [7–10]. ACP is an evidence-based practice that reduces suffering and improves QoL through patient-centered shared decision-making that incorporates patients’ values and life goals into care decisions, ideally for the duration of the life-limiting illness, but at a minimum near the end of life [11–15].

This study aimed to identify barriers and facilitators to effective implementation of Shared Decision Making-Renal Supportive Care (SDM-RSC), an intervention to improve ACP for patients with ESKD on hemodialysis who were estimated to be in the last 6 months of life [16]. The goal of this study was to inform future efforts to scale-up the SDM-RSC intervention for use in non-research clinical settings.

## Methods

The methods for the SDM-RSC intervention study are described first below to provide context for the current study. The methods for the current study follow the brief description of the SDM-RSC study.

### The intervention

The SDM-RSC intervention sought to increase use of evidence-based ACP practices [17, 18] for patients with ESKD on hemodialysis in Massachusetts (*n* = 10 dialysis units) and New Mexico (*n* = 8 dialysis units) who were estimated to be in the last 6 months of life based on a validated prognostic tool [19]. Development of the SDM-RSC intervention was guided by Stakeholder and Patient Advisory Boards, dialysis staff, and formative interviews with patients [20]. The Stakeholder Advisory Board included eight members with representation from national leaders in social work and renal palliative care and from proprietary dialysis chain leadership (chief medical officers and medical directors). The Patient Advisory Boards (one in New Mexico, and one in Massachusetts) included a total of 17 patients on dialysis and family members of dialysis recipients. The Advisory Boards provided guidance and feedback to the research team over the course of the study; the Boards met approximately monthly, separate from the research team, in the first 6 months of the study, then approximately twice yearly to respond to inquiries from the research team. A chairperson from the Advisory Boards also attended research team meetings. Additional details of the intervention protocol are available in Additional file 1: Appendix A and full details have been published elsewhere [16]. Briefly, social worker ACP training included an introduction to the rationale for the study and a full day of didactic lessons that was led by four social workers, a lawyer, and a palliative care physician. The didactic training curriculum was developed using existing literature and resources, data from focus groups conducted with the social workers in the two study regions, and input from the study’s Advisory Boards. The training team provided four additional telephonic “booster” sessions for social workers over 2 years. Nephrologists participated in a one-hour training session that included an introduction to the rationale for the study, review of mortality rates for hemodialysis patients, review of the literature on ACP for hemodialysis patients, tools for conducting ACP discussions, and a video demonstrating key elements of ACP discussions.

After determining which patients would be eligible to participate in the study, research staff worked with dialysis unit social workers to approach eligible patients in the dialysis units to invite them to participate, to answer questions, obtain informed consent, collect baseline data, and schedule the ACP discussion. If the patient wanted a family member to be involved the patient was invited to bring them to the ACP discussion. If the patient had a surrogate, the research team member responsible for recruitment discussed the study them by phone after introduction by the dialysis unit social worker. Social worker-nephrologist teams led ACP discussions with patients or their surrogate from their dialysis units who were enrolled in the study; with family members were present if the patient desired. Participant preferences for discussing prognosis, life goals, and goals of care were elicited in a pre-intervention questionnaire; these data were available to the social worker and nephrologist prior to the ACP session. Social workers conducted follow-up conversations with the participant and/or family members as indicated by the discussion in the initial ACP session. ACP sessions took place between February 2015 and March 2017. The study protocol was approved by the Institutional Review Boards at the University of Massachusetts Medical School-Baystate and the University of New Mexico.

### Barriers and facilitators to implementation

Data were collected via the following: direct observation of study procedures by research staff in the course of conducting the study; surveys of social workers, nephrologists, study participants, and their family members; and audio-recordings of initial SDM-RSC intervention sessions.

### Observations

The SDM-RSC research coordinators, research assistants, co-principal investigators, and co-investigators were asked to identify factors that they felt might facilitate or hinder implementation of the study procedures during pre-intervention, recruitment, and intervention phases of the study. Reports of observations were elicited during weekly research team meetings and documented in meeting minutes. The Advisory Boards were also asked to also identify perceived potential facilitators and barriers to implementation, which were communicated by Advisory Board leaders during research team meetings and documented in meeting minutes.

### Surveys

Social workers, nephrologists, participants, and families were asked to complete questionnaires (Additional file 2: Appendix B) that elicited feedback about the intervention (Table 1). The questionnaires were developed by the study’s principal investigators, co-investigators, and research staff and were reviewed by the Advisory Boards for clarity and completeness. Briefly, social workers who participated in an ACP session were asked to complete monthly surveys by phone after they had conducted their first ACP session. The questionnaires assessed their experience with conducting the ACP sessions and elicited feedback for improvements. Study participants and family members who attended an ACP session completed questionnaires between 1 to 3 days following the initial ACP session. These questionnaires assessed satisfaction with the ACP session and elicited feedback for improvement. Finally, nephrologists completed a questionnaire at the end of the study that included both open-ended and Likert-scale questions that assessed their experience conducting the ACP intervention and satisfaction with the intervention design and implementation (Additional file 2: Appendix B).

**Table 1:** Content of Surveys Evaluating SDM-RSC Intervention

Survey	Key Content	Timing of Survey
Social worker monthly assessment	Satisfaction with the intervention Recommendations for future efforts to implement	Monthly following social worker first SDM-RSC intervention session
Nephrologist post-study assessment	Satisfaction with the intervention Recommendations for future efforts to implement	End of the intervention phase of the study
Participant-Patient post-intervention assessment	Satisfaction with the intervention	1 to 3 days following intervention session
Participant-Family post-intervention assessment	Satisfaction with the intervention	1 to 3 days following intervention session

### Fidelity assessment

A random sample of 20% of the SDM-RSC intervention sessions were audio-recorded with patient, family member, nephrologist, and social worker permission. Two research team members (SG and NE) assessed intervention fidelity by determining whether the following elements of ACP were present: 1) The social worker began the session with introductions; 2) The patient’s health situation was discussed; 3) There was an effort to elicit the patient’s life goals; 4) Prognosis was discussed; 5) The nephrologist or social worker summarized the discussion; 6) The patient/family were informed that the social worker would follow-up with them individually if they wished; 7) The patient was asked to summarize what was discussed and decided during the meeting; and 8) Patient/family members were given an opportunity to ask questions. The elements of fidelity assessed were derived from training materials for the SDM-RSC intervention. Nephrologists and social workers were given access to the fidelity checklist prior to holding ACP discussions with study participants.

### Analyses

#### Observations and open-ended survey questions

The lead author (SG) conducted thematic analysis of direct observations and responses to open-ended survey questions to identify potential barriers and facilitators to implementing the ACP intervention [25]. Thematic analysis is a systematic approach to identifying patterns or “themes” in qualitative data [23]. Data from the two sources were combined to capture the multiple perspectives represented. This study’s lead author read all meeting minutes and open-ended survey responses, creating a code book using the Consolidated Framework for Implementation Research (CFIR) [26] as an analytic framework. The CFIR is a menu of factors that have been associated with effective implementation across five constructs, each with associated domains (examples of domains are listed after each construct): Intervention Characteristics (complexity, adaptability); Outer Setting (external and internal incentives); Inner Setting (culture, tension for change, learning climate); Characteristics of Individuals (knowledge and beliefs about the intervention, self-efficacy); and Process (opinion leaders, champions, planning, engaging) (Additional file 3: Appendix C).

#### Surveys

Descriptive statistics (e.g., numbers and percents) were used to report overall survey results. Average scores with standard deviations were calculated for Likert-style survey questions.

#### Fidelity assessment

Two study team members (SG and NE) conducted fidelity assessments of the first four audio-recordings of the SDM-RSC intervention sessions independently and discussed differences in scoring to develop consistency in assessments. The remaining audio-recordings were evaluated independently by SG and NE; four additional audio-recordings were assessed by both SG and NE mid-way through the intervention period to ensure that consistency was maintained. Descriptive statistics (numbers and percents) were used to characterize the proportion of interventions sessions recorded that contained each of the eight elements assessed.

## Results

A total of 125 hemodialysis patients (65 in MA and 60 in NM) and 47 family members (17 in MA and 30 in NM) were enrolled in the SDM-RSC study (Table 2). 102 SDM-RSC intervention sessions were conducted: 58 with patients alone, 40 with patients and family members present, and four with a family member or surrogate alone. Ninety-four patients and 42 family members completed post-intervention questionnaires.

**Table 2:** Patient Characteristics

Characteristic	% (n)
Gender	
Female	49% (61)
Male	51% (64)
Age (years)	
18–64	31% (39)
65+	69% (86)
Race, Ethnicity	
American Indian	12% (15)
Asian	1% (1)
Native Hawaiian	0% (0)
Black or African American	14% (18)
White	46% (57)
More than 1 race	4% (5)
Unknown or Other	23% (21)
Ethnicity	
Hispanic or Latino	37% (46)
Not Hispanic or Latino	62% (78)
Unknown or not reported	1% (1)
Education (*N* = 124)	
<High school education	22% (22)
Graduated	32% (40)
Some college	28% (35)
College graduate	18% (23)
Medical History (*N* = 120)	
History of myocardial infarction	23% (24)
History of diabetes	73% (87)

A total of 29 nephrologists (20 MA and 9 NM) and 24 social workers (9 MA and 15 NM) were trained to conduct ACP intervention sessions. A total of 108 monthly surveys were completed by social workers and 20 nephrologists completed the post-study survey. Examples of potential barriers and facilitators were identified in each of the five CFIR domains (Intervention Characteristics, Outer Setting, Inner Setting, Personal Characteristics, and Process) from at least one of the data sources: observations and surveys – quantitative; observations and surveys – qualitative; or audio-recordings of the SDM-RSC intervention sessions. Representative quotes for the qualitative analysis are included in Table 3.

**Table 3:** Representative Quotes: Social Worker and Nephrologist Responses to Open-Ended Survey Questions

	Sample Quotes from Surveys
What Worked Well	
Intervention Characteristics	Put[s] the patient in control [of the discussion], not like rounds (SW) [Provided] an opportunity to talk with family and patient together (SW) Fostered trust between the patient and the team (SW)
Process	Having patient’s pre-survey ahead of time Intervention guideline (SW) E-mail reminders about what to cover (SW) Having privacy of an isolation room if ACP discussion took place while patient on dialysis (SW)
What Did Not Work Well	
Intervention Characteristics	SW must be [physically] present at ACP discussion [not on phone] (SW)
Process	Families and patients needed more preparation for ACP discussion (SW) Doctor and social worker need to meet before ACP discussion to coordinate roles (SW) Too much time between recruitment and intervention (SW) Not good to have ACP discussion while patient on dialysis (SW)
Inner Setting	Hard to schedule time when all can be there (SW) Recording intervention session felt awkward (SW)
Outer Setting	Some patients not ready to discuss ACP (SW) Difficulty conducting discussion with patients with limited English proficiency (SW)
Personal Characteristics	None – the intervention is not necessary
Recommendations for Changes	
Process	Start ACP either as soon as a patient begins or just prior to beginning hemodialysis Have more frequent follow-up discussions after initial discussion (SW) Meet patients at their homes for ACP discussions (MD) Conduct ACP discussions during rounds (MD) Have hospice worker or hospice handout at ACP discussion (SW) Train all dialysis staff to talk about ACP (MD) Make training inter-professional (SW)
Inner Setting	Make it a policy to hold ACP discussions annually (SW)
Outer Setting	Pay SW more for the additional work (MD)
Personal Characteristics	None – the intervention is not necessary (SW)

*SW* Social Worker, *MD* Nephrologist

### Intervention characteristics

#### Surveys - quantitative

Patients and family members reported high levels of comfort (1 = very comfortable; 4 = very uncomfortable) with the intervention with mean ratings of 1.54 (SD = 1.12) by patients and 1.19 (SD = 0.51) by family members.

#### Surveys - qualitative

Two patients and family members reported experiencing emotional distress in response to the intervention on open-ended survey responses. Conversely, other patients and family members expressed a preference for more specific discussion about prognosis,“[I] wanted to know how long I have to live, but they said they couldn’t pinpoint it.”

Social workers’ responses to open-ended survey questions about the intervention included feeling that the intervention promoted interdisciplinary teamwork and that the intervention put patients in control of the discussion. Nephrologists’ open-ended survey responses were brief: the majority expressed satisfaction with the intervention overall, but they also expressed concerns about the feasibility of conducting ACP discussions with all patients due to lack of time.

### Outer setting

#### Observations - qualitative

In the process of refining study procedures prior to the start of the study, the research team learned that hemodialysis is considered a life-sustaining treatment by most hospices, and as such disqualifies patients from receiving hospice services unless they have a life-limiting illness other than ESKD. Since mean survival after withdrawal of dialysis is 7.4 days (range 0 to 40) [27], many participants in the study could only be enrolled in hospice at the very end of life. Although ACP could still take place, patients who may have benefitted from hospice but did not want to withdraw hemodialysis had fewer options for end-of-life (EoL) care compared to patients with other life-limiting illnesses.

### Inner setting

#### Observations and surveys - qualitative

Research assistants’ direct observations identified lack of clinician time as a potential barrier to effective implementation of the SDM-RSC intervention; lack of time was also reported as a barrier by both nephrologists and social workers in responses to open-ended survey questions. Research team members reported as an observation that they experienced difficulties in scheduling the ACP sessions because of clinicians’ busy schedules and some nephrologist-social worker teams reported on surveys that more time was needed for the ACP sessions. Some nephrologists expressed concerns on open-ended survey responses that the unpredictable nature of patients’ emotional responses to discussions about EoL made it infeasible to plan on a circumscribed time for the ACP sessions. Although the clinicians did not comment directly on the role health care finance and organization may have on their limited available time, these factors may represent an Outer Setting barrier to implementing the SDM-RSC intervention in non-research settings.

In addition to lack of time, finding an appropriate space in which to conduct intervention sessions was also identified by some social workers and nephrologists as a barrier in surveys and as an observation by the research staff. Communication of the study goals and logistics of implementing the intervention across multiple dialysis units and in two different states were also noted to be potential barriers in observations made by the research staff.

### Personal characteristics

#### Observations and surveys - qualitative

Observations by the research staff identified Personal Characteristics as a potential barrier and facilitator to effective implementation of the SDM-RSC intervention. For example, research staff reported that one social worker did not feel it was appropriate to refer hemodialysis patients to hospice and declined to participate in the study. In contrast, they reported that several social workers became ‘champions’ for the intervention and worked closely with them to recruit patients and to facilitate implementation of the study procedures. Some social workers commented in response to open-ended survey questions that the intervention empowered them to make use of their communication skills and ability to manage social matters and that the intervention elevated the value of these skills within the dialysis team.

### Process

#### Observations and surveys- qualitative

Both nephrologists and social workers indicated on open-ended survey responses that they found having information about an individual patient’s preferences and goals prior to the intervention session useful. However, they also indicated that having this information even earlier could be more helpful. Nephrologists and social workers also indicated on surveys that they found the “reminder sheet” provided by the research team that listed the key elements to include in the intervention sessions helpful. Social workers recommended on surveys that a hospice worker be present at the intervention sessions, or that written materials about hospice be made available for patients and their family members during the sessions.

Social workers reported on the survey that they felt the intervention would be improved if all dialysis workers received ACP training and if training were interprofessional rather than conducted separately.

Nephrologists indicated on surveys that they felt that the involvement of national dialysis chain leaders as advisors to the study facilitated implementation of the study because of “buy-in” from leadership.

#### Fidelity assessment

Twenty-three of the 98 SDM-RSC intervention sessions (23.4%) at which a patient was present were recorded (7/59 in MA; 16/43 in NM). While patients and families were reported by research staff to be willing to have ACP sessions recorded, some participating nephrologists and social workers declined to give permission for recording. Recorded intervention sessions included a median of five (of eight) elements assessed. Less than half of the intervention sessions included a discussion of prognosis or a request that the patient explain their understanding of what had been covered in the meeting (47.8% each).

## Discussion

This study identified potential barriers and facilitators to consider in future efforts to scale-up the SDM-RSC intervention to improve ACP for patients with ESKD on hemodialysis. The barriers and facilitators, which were categorized using the CFIR framework’s implementation domains [26], also fit into the social ecological model of health [28]. This model demonstrates the interconnectedness of healthcare organizations, the people who work within them, the patients they serve, the communities they are situated in, and the national socio-political milieu in which they are embedded. Efforts to scale-up the SDM-RSC will need to take this interconnectedness into account.

The majority of ACP studies conducted with patients with ESKD on hemodialysis and patients with other life-limiting illnesses have taken place in controlled research settings in which the interventions were either fully or partially conducted by research team personnel [22, 29]. In the current study, the research team partnered dialysis units’ nephrologists, social workers, and patients to refine and carry out the intervention. However, despite this high level of engagement of end-users in development and implementation of the SDM-RSC intervention, the current study suggested that future efforts to scale-up and implement the intervention would benefit from even more involvement of end-users in planning and implementation.

The ACP discussions conducted during the SDM-RSC intervention sought to elicit patient’s goals of care in the context of their values and life goals. This approach contrasts with ACP discussions that primarily emphasize completion of advance directive forms, such as the Medical Orders for Life Sustaining Treatment [24, 29]. Although the comparative effectiveness of these approaches was not part of the current study, prior studies identified differences in clinicians’ beliefs about the objective of ACP discussions, including the importance of discussing prognosis [21]. Although clinicians’ reasons for not discussing prognosis were not systematically assessed in the current study, the reasons why prognosis was discussed in just under half of the SDM-RSC intervention sessions may have been due to similarly different beliefs. The failure to discuss prognosis also might have been due to patient preference, clinicians’ discomfort in discussing prognosis, or other factors. Understanding nephrologists’, social workers’, and dialysis staff’s attitudes and beliefs regarding the role of discussing prognosis during ACP is an important consideration for future implementation of the SDM-RSC intervention.

Some of the barriers to implementation of the SDM-RSC intervention identified in the current study related to processes that could potentially be modified at the organizational level (e.g., better patient/family preparation prior to ACP discussions and streamlining the implementation process), but some would be best addressed at the level of health policy related to financing and delivery of health care. For example, the lack of sufficient time and facilities for conducting ACP discussions has been identified as a barrier in other studies of ACP for patients with life-limiting illnesses [30]. This barrier might be mitigated by value-based financial models, which, in theory, enable clinical teams to deliver care in a more flexible way than fee-for-service models. Also, policies that allow patients to receive hospice care while continuing dialysis would increase the options for palliative care that can be offered during ACP discussions. These high-level changes will require those in a position to serve as advocates for patients with ESKD on hemodialysis to work with policy-makers at the national level to bring about these changes.

## Conclusions

This study suggests that future efforts to scale-up and implement the SDM-RSC intervention could benefit from additional ACP training for both social workers and nephrologists, including interprofessional training. This study also suggests that some of the barriers identified may be obviated by involving local clinicians, staff, dialysis patients, and their families in decisions about processes for conducting ACP discussions at an early stage of implementation of the intervention. The impact of healthcare policies, such as those that may contribute to a perceived lack of time for ACP discussions in current work flows and challenges to accessing hospice services while on hemodialysis should also be considered if ACP is to become a routine practice for healthcare providers and their patients facing the high morbidity and mortality associated with hemodialysis.

## Additional files

Additional file 1: Appendix A: SDM-RSC Intervention Description. This file provides additional details of the parent study intervention. (DOCX 15 kb)

Additional file 2: Appendix B: Questionnaires. This file contains copies of the questionnaires used to evaluate implementation of the study. (DOCX 13 kb)

Additional file 3: Appendix C: Consolidated Framework for Implementation Research Domains [31]. This file contains a description of Constructs and Domains of the CFIR Framework. (DOCX 19 kb)

## Data Availability

The datasets used and/or analyzed during the current study are available from the corresponding author on reasonable request.

## Abbreviations

ACP: Advance Care Planning
CFIR: Consolidated Framework for Implementation Research
EoL: End of Life
ESKD: End Stage Kidney Disease
QoL: Quality of Life
SDM-RSC: Shared Decision-Making – Renal Supportive Care
SPIRIT: Sharing Patient’s Illness Representations to Increase Trust
