# Supplementary material for: Advance care planning with patients on hemodialysis: an implementation study
Source: BMC Palliat Care. 2019 Jul 26;18:64. doi: 10.1186/s12904-019-0437-2 (PMC6659207; doi:10.1186/s12904-019-0437-2)
Supplement: Supplementary file 1 — Appendix A: SDM-RSC Intervention Description. This file provides additional details of the parent study intervention. (DOCX 15 kb) [file 12904_2019_437_MOESM1_ESM.docx]

**Appendix A: SDM-RSC Intervention Description** (16)

The SDM-RSC study was a multi-center study designed to test the effectiveness of an intervention to improve Advance Care Planning (ACP) for patients with ESKD receiving hemodialysis and who are at high risk of death in the ensuing six months. In the multi-modal intervention, nephrologists and social workers communicated prognosis and provided ACP during face-to-face encounters with patients and families using an algorithm that situated dialysis unit social workers at the center of the intervention. Follow-up sessions with the social worker took place monthly following the initial discussion if desired by the participant to provide further support, education, information, and referral to resources such as hospice.

Patients were recruited from dialysis units associated with two large academic centers; ten units were affiliated with Baystate Medical Center in Springfield, Massachusetts and eight were affiliated with the University of New Mexico in Albuquerque, New Mexico.

Prognosis was determined for all patients attending the study dialysis clinics using a previously developed prognostic tool. Predictors of time to death were age, serum albumin, absence/presence of dementia, absence/presence of peripheral vascular disease and a modified Surprise Question (SQ). The SQ asks nephrologists “Would you be surprised if this patient died during the next six months?” Patients who were receiving hemodialysis and were: 1) English or Spanish-speaking, 2)  18 years of age or older, and 3) who were in the highest quartile of predicted mortality risk were eligible for participation in the study. Exclusion criteria included: 1) having a diagnosis of a severe psychiatric disorder (i.e. schizophrenia, bipolar disorder, other psychiatric disorder warranting hospitalization in the past month), 2) expectation of native kidney function recovery, 3) scheduled for living donor kidney transplant, 4) history of poor adherence to hemodialysis treatments (i.e. missing ≥ 4 treatments in the last month), and 5) exclusion by the primary nephrologist or social worker due to risk of harm. Children under18 years of age were not eligible for study participation since the physical factors related to ESKD for children are not directly comparable to those of adults. Patients who lacked capacity to meaningfully participate in medical decisions were eligible for inclusion if they had a surrogate who was interested in participating in the study and was eligible to provide written informed consent.

Baseline data were collected for patients and caregivers at the time of enrollment. The initial patient and caregiver ACP discussion took place with the nephrologist responsible for managing the patient’s dialysis and the social worker from the patient’s dialysis unit. Patient and caregiverpreferences for prognostic and EoL discussions were broached in the meeting. Feedback on the intervention was collected throughout the study (see Table 1). Additional data about hospice referrals was collected from administrative data. Patient flow was tracked to capture those who were lost to follow-up.

The primary outcome for this intervention was the timing and percentage of hospice referrals. This was determined through administrative data, as well as through interviews with bereaved family members and the dialysis staff. A secondary outcome was the location of the participant’s death (i.e., home, hospital, intensive care unit, or nursing home) ascertained through the interviews with bereaved families and the dialysis social workers who cared for the patient prior to death. An additional secondary outcome was completion of advanced directive documents. Caregivers were also asked to respond to questions about the quality of dying. Instruments used for data collection included a measure of health-related quality of life (HRQoL), pain, depression symptoms, satisfaction with care, and caregiver distress.
