# Supplementary material for: Advance care planning with patients on hemodialysis: an implementation study
Source: BMC Palliat Care. 2019 Jul 26;18:64. doi: 10.1186/s12904-019-0437-2 (PMC6659207; doi:10.1186/s12904-019-0437-2)
Supplement: Supplementary file 2 — Appendix B: Questionnaires. This file contains copies of the questionnaires used to evaluate implementation of the study. (DOCX 13 kb) [file 12904_2019_437_MOESM2_ESM.docx]

**Appendix B: Questionnaires**

**1. SW Opinion Survey (Staff - Phone)**

This will be administered to all of the social workers who participated in the study intervention. It will be given monthly or if one of these staff people leave earlier for another position involving a dialysis clinic that is not involved in the research.

All questions are since last phone call or in the last 30 days

1. We are interested in your experience of the SDM-RSC intervention, beginning with the initial meeting with patients and/or families. How did the meetings go?

2. What aspects worked well?
3. What aspects did not work well?

4. How was the Information Sheet received?

5. What unexpected things (good or bad) occurred during the meetings? What things (good or bad) occurred during the subsequent follow-up experiences with patients and/or families?

6. How were the subsequent meetings between the social worker and patient received?

7. How would you rate your satisfaction with the SDM-RSC intervention (including the initial meeting and the follow-up contacts of the social worker)?

8. How would you rate the impact of the study's intervention from the perspective of your patients/families?

9. What suggestions do you have to improve the SDM-RSC intervention (including the initial meeting and the follow-up contacts of the social worker), if they are to be used nationally?

10. Do you anticipate continuing the intervention at your clinic?

11. Do you have any further comments for the researchers?

**2. Final Staff Nephrologist QA Opinion Survey (Staff - Phone)**

This will be administered to all of the Nephrologists who participated in the study intervention. It will be given at the conclusion of the study or if one of these staff people leave earlier for another position involving a dialysis clinic that is not involved in the research.

1. We are interested in your experience of the SDM-RSC intervention, beginning with the initial meeting with patients and/or families. How did the meetings go?

2. What aspects worked well?

3. What aspects did not work well?

4. How was the Information Sheet received?

5. What unexpected things (good or bad) occurred during the meetings? What things (good or bad) occurred during subsequent follow-up experiences with patients and/or families?

6. How were the subsequent meetings between the social worker and patient received?

7. How would you rate your satisfaction with the SDM-RSC intervention (including the initial meeting and the follow-up contacts of the social worker?

8. How would you rate the impact of the study's intervention from the perspective of your patients/families?

9. What suggestions do you have to improve the SDM-RSC intervention (including the initial meeting and the follow-up contacts of the social worker), if they are to be used nationally?

10. Do you anticipate continuing the intervention at your clinic?

11. Any further comments for the researchers?

**3. Post-Initial Meeting Questions For Patients (Patient - Phone)**

Either of these sets of questions would be administered to the patient by the 24-72 hours following intervention (i.e., at next dialysis session or by telephone). The subjects are again to be instructed that we would prefer that their answers be frank, honest, and reflect their realistic opinions.

SCRIPT: For each question, I'd like you to be as frank and honest as you can.

1. On a scale of 1 through 5, how comfortable were 1 you with the meeting you (and your family member) 2 just had with the nephrologist and dialysis social 3 worker? 1 is very comfortable, and 5 is very 4 uncomfortable. 5

2. On a scale of 1 through 5, did you find the 1 meeting to have been distressing? 1 is very 2 distressing, and 5 is not distressing. 3

3. We are interested in hearing your comments:

4. Do you have any comments on the meeting that you (and your family member) had with the social worker and the nephrologist?

**4. Post-Initial Meeting Questions For Families (Family - Phone)**

This form will be telephone administered to the family 24-72 hours by the CRA or CRC. The subjects are to be instructed that we would prefer that their answers be frank, honest, and reflect their realistic opinions.

SCRIPT: I'm going to ask you a few questions about the meeting you had with your family members, doctor and social worker. I'd like to ask you those questions first. For each of them, I'd like you to be as frank and honest as you can.

1. On a scale of 1 through 5, how comfortable were 1 you with the meeting you (and your family member) 2 just had with the nephrologist and dialysis social 3 worker? 1 is very comfortable, and 5 is very 4 uncomfortable. 5

2. On a scale of 1 through 5, did you find the 1 meeting to have been distressing? 1 is very 2 distressing, and 5 is not distressing.

3. We are interested in hearing your comments:

4. Do you have any other comments on the meeting that you (and your family member) had with the social worker and the nephrologist?
