# Supplementary material for: Advance care planning with patients on hemodialysis: an implementation study
Source: BMC Palliat Care. 2019 Jul 26;18:64. doi: 10.1186/s12904-019-0437-2 (PMC6659207; doi:10.1186/s12904-019-0437-2)
Supplement: Supplementary file 3 — Appendix C: Consolidated Framework for Implementation Research Domains [31]. This file contains a description of Constructs and Domains of the CFIR Framework. (DOCX 19 kb) [file 12904_2019_437_MOESM3_ESM.docx]

**Appendix C: Consolidated Framework for Implementation Research Domains (1)**

| **Domain** | **Examples** |
| --- | --- |
| **Outer Setting** | A broad construct that includes external strategies to spread interventions, including policy and regulations (governmental or other central entity), external mandates, recommendations and guidelines, pay-for-performance, collaboratives, and public or benchmark reporting |
| **Inner Setting** | The nature and quality of webs of social networks and the nature and quality of formal and informal communications within an organization.  The absorptive capacity for change, shared receptivity of involved individuals to an intervention, and the extent to which use of that intervention will be rewarded, supported, and expected within their organization.  The degree to which stakeholders perceive the current situation as intolerable or needing change.  The degree of tangible fit between meaning and values attached to the intervention by involved individuals, how those align with individuals’ own norms, values, and perceived risks and needs, and how the intervention fits with existing workflows and systems.  The degree to which goals are clearly communicated, acted upon, and fed back to staff, and alignment of that feedback with goals  Commitment, involvement, and accountability of leaders and managers with the implementation.  Ease of access to digestible information and knowledge about the intervention and how to incorporate it into work tasks. |
| **Characteristics of Individuals** | Individuals’ attitudes toward and value placed on the intervention as well as familiarity with facts, truths, and principles related to the intervention.  Individual belief in their own capabilities to execute courses of action to achieve implementation goals.  A broad construct related to how individuals perceive the organization, and their relationship and degree of commitment with that organization.  A broad construct to include other personal traits such as tolerance of ambiguity, intellectual ability, motivation, values, competence, capacity, and learning style. |
| **Process** | The degree to which a scheme or method of behavior and tasks for implementing an intervention are developed in advance, and the quality of those schemes or methods.  Attracting and involving appropriate individuals in the implementation and use of the intervention through a combined strategy of social marketing, education, role modeling, training, and other similar activities.  Individuals in an organization who have formal or informal influence on the attitudes and beliefs of their colleagues with respect to implementing the intervention.  Individuals from within the organization who have been formally appointed with responsibility for implementing an intervention as coordinator, project manager, team leader, or other similar role.  Individuals who dedicate themselves to supporting, marketing, and ‘driving through’ an implementation, overcoming indifference or resistance that the intervention may provoke in an organization.  Individuals who are affiliated with an outside entity who formally influence or facilitate intervention decisions in a desirable direction.  Carrying out or accomplishing the implementation according to plan.  Quantitative and qualitative feedback about the progress and quality of implementation accompanied with regular personal and team debriefing about progress and experience. |
| **Intervention** | Perception of key stakeholders about whether the intervention is externally or internally developed.  Stakeholders’ perceptions of the quality and validity of evidence supporting the belief that the intervention will have desired outcomes.  Stakeholders’ perception of the advantage of implementing the intervention versus an alternative solution.  The degree to which an intervention can be adapted, tailored, refined, or reinvented to meet local needs.  Perceived difficulty of implementation, reflected by duration, scope, radicalness, disruptiveness, centrality, and intricacy and number of steps required to implement.  Perceived excellence in how the intervention is bundled, presented, and assembled. |

1. Damschroder LJ, Aron DC, Keith RE, Kirsh SR, Alexander JA, Lowery JC. Fostering implementation of health services research findings into practice: a consolidated framework for advancing implementation science. Implement Sci IS. 2009 Aug 7;4:50.
